# Laparoscopic Management of Bouveret’s Syndrome: A Surgical Case Series

**DOI:** 10.7759/cureus.95579

**Published:** 2025-10-28

**Authors:** Iman Hameed, Aaron Rockliff, Nezor Houli

**Affiliations:** 1 General Surgery, Western Health, Footscray, AUS; 2 Upper Gastrointestinal Surgery, Western Health, Melbourne, AUS

**Keywords:** bouveret’s syndrome, cholecystoduodenal fistula, gallstone disease, gallstone ileus, minimally invasive surgery

## Abstract

Bouveret’s syndrome is a form of gastric outlet obstruction characterised by gallstones traversing through a cholecystoduodenal fistula and lodging in the proximal duodenum and pylorus. This condition often requires complex surgical management due to disruption of the normal biliary and duodenal anatomy. The management approach is usually tailored to the patient’s disease, the surgeon’s and endoscopist’s skills and instrument availability. The principles for treatment are ectopic gallstone extraction, management of duodenotomy, cholecystectomy and repair of cholecystoduodenal fistula.

This case series presents three instances of Bouveret’s syndrome, each involving gallstone impaction at a distinct location within the duodenum. All cases were performed in single-stage procedures. We have included a summary of the operative steps and described our techniques in detail.

All patients underwent successful laparoscopic surgery with uneventful recovery, no reoperation, and no evidence of fistula recurrence or stricture on follow-up.

Although this condition is rare, general surgeons should be familiar with the principles of managing a cholecystoduodenal fistula and seek early specialist input, as it carries a high risk of morbidity and mortality. Where feasible, a single-stage, minimally invasive approach should be considered, as it may reduce the need for multiple operations and improve overall outcomes.

## Introduction

Bouveret’s syndrome represents a rare variant of gastric outlet obstruction resulting from the passage of a gallstone through a cholecystoduodenal fistula, leading to impaction within the duodenum or pylorus. This condition is seen in less than 0.5% of all gallstone disease [1] and results in gastric outlet obstruction with concurrent cholecystitis, choledocholithiasis or cholangitis. It also carries a high risk of morbidity and mortality associated with surgery, up to 25% [1]. Patients will typically present with non-specific symptoms including epigastric pain, nausea, profuse vomiting and fever. The presence of Rigler’s triad of pneumobilia, gastrointestinal obstruction and ectopic gallstones on imaging supports this diagnosis. The management approach is usually tailored to the patient’s disease, the surgeon’s and endoscopist’s skills and equipment availability. For context, all our cases were operated and managed by a combination of general, upper gastrointestinal and hepatobiliary surgeons.

## Case presentation

Case 1

A 69-year-old female patient presented with a one-day history of upper abdominal pain associated with nausea, vomiting and fever.

The patient’s past medical history included morbid obesity with a BMI of 68, previous lower uterine caesarean section (LUCS) and symptomatic gallstones. Her blood results showed a mild CRP rise with no signs of biliary obstruction [1]. These are demonstrated in Table 1.

**Table 1:** Case 1 blood test results WCC: white cell count, ALT: alanine aminotransferase, AST: aspartate aminotransferase, ALP: alkaline phosphatase, GGT: gamma-glutamyl transferase

Blood test	Value	Normal range [2]
WCC	10.8	3.5-11.0 x 10^9/L
CRP	41	<3.0mg/L
Bilirubin	11	<20.0 mg/dL
ALT	15	<51 U/L
AST	25	<36 U/L
ALP	142	30-110 U/L
GGT	61	5-50 U/L

**Figure 1:**
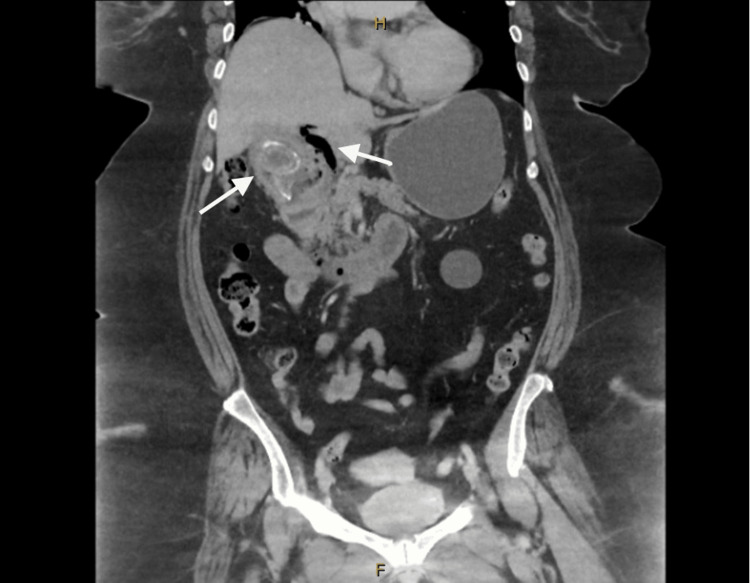
CTAP with IV contrast slices demonstrating 4 cm gallstones and pneumobilia in the left intrahepatic bile duct (Case 1) CTAP: computed tomography abdomen pelvis, IV: intravenous

On examination, her abdomen was soft with very mild right upper quadrant tenderness. Computed tomography abdomen pelvis (CTAP) with Intravenous (IV) contrast was performed, demonstrating two large calculi in the gallbladder lumen, with the largest measuring 4 x 3 cm (Figure 1).

**Figure 2:**
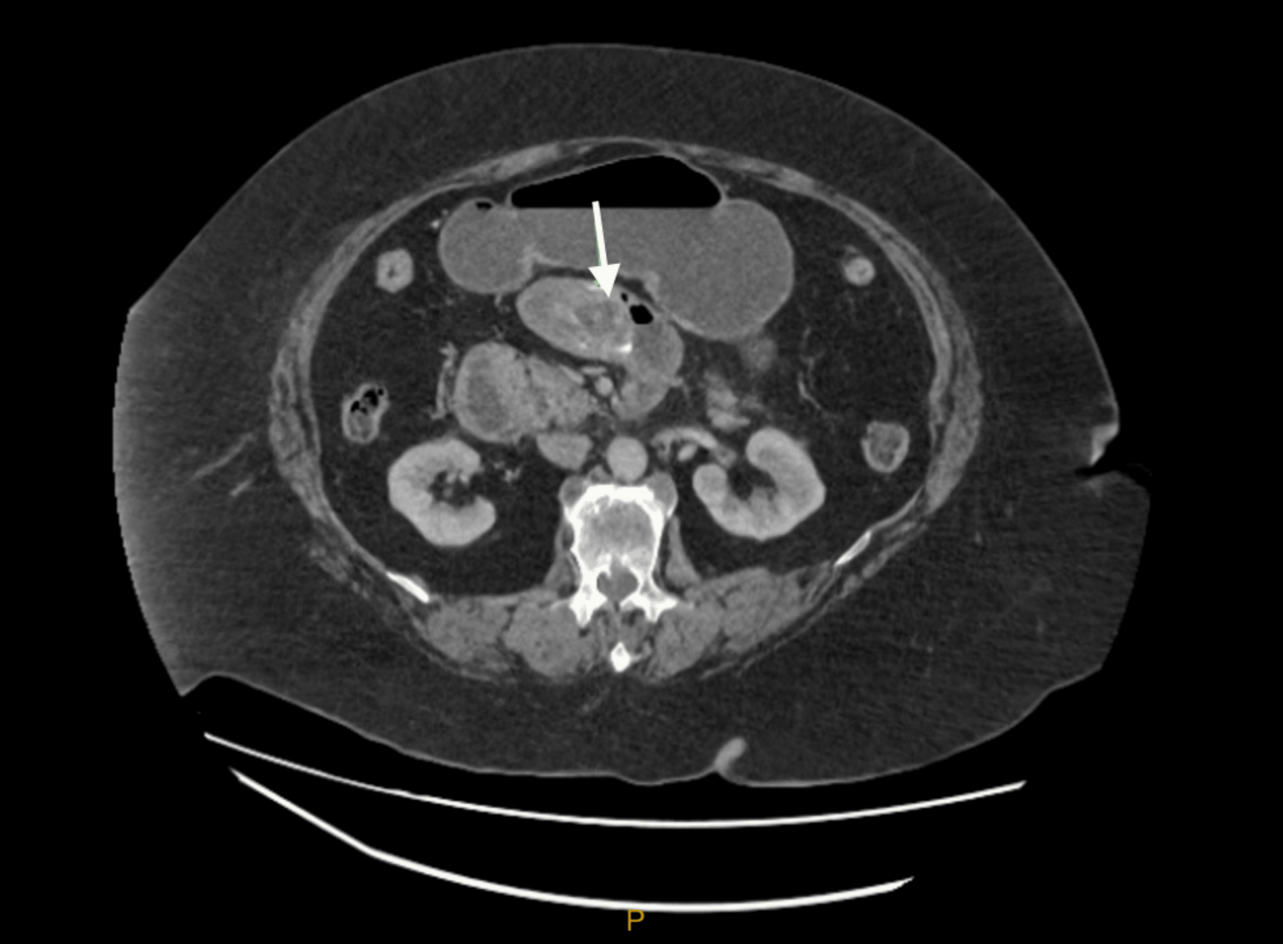
Additional 4 cm gallstone in distal duodenum

Additionally, there was a gallstone measuring 4 x 2 cm in the distal duodenum anterior to the proximal superior mesenteric vein, which was compressed by the stone, and partial duodenal obstruction at the level of the duodenojejunal flexure (Figure 2). The stomach and duodenum were distended with diffuse thickening of D2 (the second part of the duodenum) and D3. There was pneumobilia in the common hepatic duct extending into the left intrahepatic duct. There was also inflammatory oedema of the gallbladder neck, which was in close proximity to the cystic duct and common bile duct. A cholecystoduodenal fistula was therefore suspected. A nasogastric tube (NGT) was inserted, and 1.2L of gastric fluid was drained. IV ceftriaxone and metronidazole were administered, and an emergency theatre was organised.

An initial gastroduodenoscopy was inserted to D4, which visualised a large stone; however, it could not be retrieved with a basket or snare. The stone was then pushed towards the proximal jejunum in preparation for enterotomy. Due to concerns about the oedema in the duodenal wall, we did not perform any further attempts at endoscopic management. We performed laparoscopy, clamping of the proximal jejunum distal to the stone and longitudinal enterotomy in the proximal jejunum. The large impacted stone was removed, and the incision was primarily repaired with 3-0 V-Loc^TM^ (Medtronic, Minneapolis, MN, USA) absorbable suture. Inspection of the gallbladder demonstrated significant chronic inflammation and inflammatory adhesions to the duodenum.

Given that the patient had a large cholelithiasis and a known fistula, the decision was made to attempt clearance of gallstones from the gallbladder. However, due to her morbid obesity, partial cholecystectomy and clearance of stones from the gallbladder were the aim rather than attempting takedown of the fistula.

An extensive adhesiolysis and subtotal cholecystectomy were performed with clearance of the two large gallbladder stones as seen on CT. A repeat endoscopy was performed, which visualised all parts of the duodenum and a fistula tract to the gallbladder. We then performed a choledochoscopy via the gallbladder into the fistula and duodenum, which confirmed a tract at the gallbladder neck. No remaining stones were seen in the gallbladder, fistula tract or proximal duodenum up to the enterotomy (stone extraction) site at proximal jejunum.

A 24Fr Foley catheter was placed as a drainage tube through the gallbladder and fistula with the tip in the duodenum. The remnant gallbladder was reconstituted with 3-0 V-Loc^TM^ absorbable suture over a 16Fr Foley catheter to prevent bile leak from stump blowout. Both catheters were tunnelled through the abdominal wall. These were placed to allow drainage, reduce the risk of leak from the gallbladder remnant and fistula tract and for future cholangiography. Subsequently, a four-quadrant washout was performed, and two subhepatic drains were inserted.

The patient was admitted to the ICU for a 24-hour period of observation, where she remained well. Postoperatively, the patient was kept nil by mouth (NBM) and maintained on total parenteral nutrition (TPN). The 16Fr Foley catheter into the gallbladder remnant was accidentally removed; however, there was no complication or leak as demonstrated with a CT oral contrast. The abdominal drains were removed prior to discharge, and the remaining Foley catheter with tip in the duodenum was removed six weeks postoperatively in the clinic.

A CT intravenous cholangiogram (CTIVC) at eight months postop showed no residual stones in the remnant gallbladder or bile ducts, and the fistula remains patent.

Summary of Operative Steps

An endoscopic removal of the ectopic gallstone was attempted but abandoned due to the stone's size. The stone was relocated to the jejunum to facilitate removal. A laparoscopic enterotomy at the proximal jejunum was performed, the gallstone was removed, and the enterotomy site was repaired. A partial cholecystectomy was performed, and the remaining stones in the gallbladder were cleared. The cholecystoduodenal fistulae were not taken down. Two gallbladder Foley catheters were used for gallbladder and fistula drainage. 

Case 2

A 60-year-old female patient presented with a one-week history of upper abdominal pain associated with bilious vomiting and diarrhoea, with a history of lymphoma that has been in remission for five years. Her exam revealed right upper quadrant tenderness with localised peritonism. Her blood results are demonstrated in Table 2.

**Table 2:** Case 2 blood test results WCC: white cell count, ALT: alanine aminotransferase, AST: aspartate aminotransferase, ALP: alkaline phosphatase, GGT: gamma-glutamyl transferase

Blood test	Value	Normal range [2]
WCC	8.2	3.5-11.0 x 10^9/L
CRP	82	<3.0mg/L
Bilirubin	10	<20.0 mg/dL
ALT	11	<51 U/L
AST	17	<36 U/L
ALP	101	30-110 U/L
GGT	39	5-50 U/L

**Figure 3:**
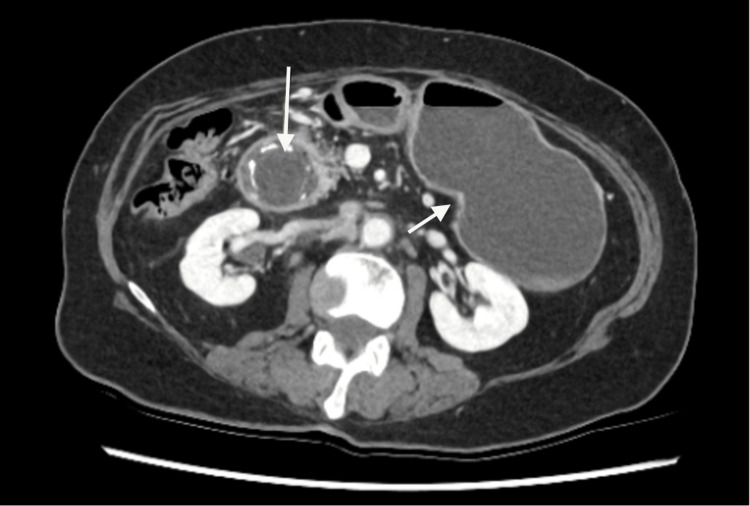
Axial slice CTAP with IV contrast demonstrating fluid filled distention of stomach and large obstructing 3 x 6 cm stone in D2 (Case 2) CTAP: computed tomography abdomen pelvis, IV: intravenous

A CTAP with IV contrast showed gastric outlet obstruction secondary to a large 3 x 6 cm obstructing calcified stone in the second part of the duodenum (D2) (Figure 3). There is marked fluid-filled distension of the stomach with associated pneumobilia, which suggests the presence of a cholecystoduodenal fistula. The patient had NGT decompression in the ED, and an urgent operation was organised.

An initial gastroduodenoscopy was performed to attempt removal of the stone; however, this was unsuccessful due to the stone's size and impaction.

An explorative diagnostic laparoscopy was then performed, which revealed significant adhesions of the hepatic flexure and omentum to the gallbladder and D1. A Cattel-Brasch manoeuvre (caudal to cranial and lateral to medial mobilisation of caecum and right colon) was performed to access D2 and D3. This technique kept the hepatic flexure adherent to the gallbladder fossa and avoided disturbing the cholecystoduodenal fistula at D1.

Following this, a longitudinal duodenotomy in D2/3 far from the ampulla and the site of the fistula was performed, and the stone was extracted. The duodenotomy was closed transversely with 3-0 V-Loc^TM ^absorbable suture.

Intraoperatively, we could not appreciate any remaining stones within the gallbladder, which correlated with the pre-operative imaging. Due to this, as well as the significant inflammation around the gallbladder, the decision was made not to disturb the cholecystoduodenal fistula or attempt removal of the gallbladder.

The patient was admitted to the ICU postoperatively for haemodynamic support. She was kept NBM for four days to allow full enteric recovery. Upon clinical improvement, slow upgrading of diet and weaning off TPN, she was discharged on Day 17.

A CTIVC performed at 10 months follow-up showed no stones and additionally no obvious residual cholecystoduodenal fistula, which indicates healing or closure of the fistula. Repeat CT at three years postop showed a shrunken gallbladder with no gas in the gallbladder, no calcified stones and no obvious soft tissue lesion to suggest a malignant lesion. The patient remains clinically well with no biliary colic and normal liver function tests (LFT).

Summary of Important Operative Steps

An endoscopic removal of the duodenal stone was attempted. A laparoscopic Cattel-Braasch manoeuvre was performed to expose D2/D3 and D4. A duodenotomy was performed, followed by clearance of a large duodenal stone and primary repair. The cholecystoduodenal fistula was not resected or explored. No cholecystectomy was performed.

Case 3

A 64-year-old male patient presented with a two-week history of epigastric pain, vomiting and inability to tolerate oral intake. This patient’s past medical history included grade 2 obesity with a BMI of 35, hypertension for which he took telmisartan, and a peptic ulcer 30 years ago.

**Figure 4:**
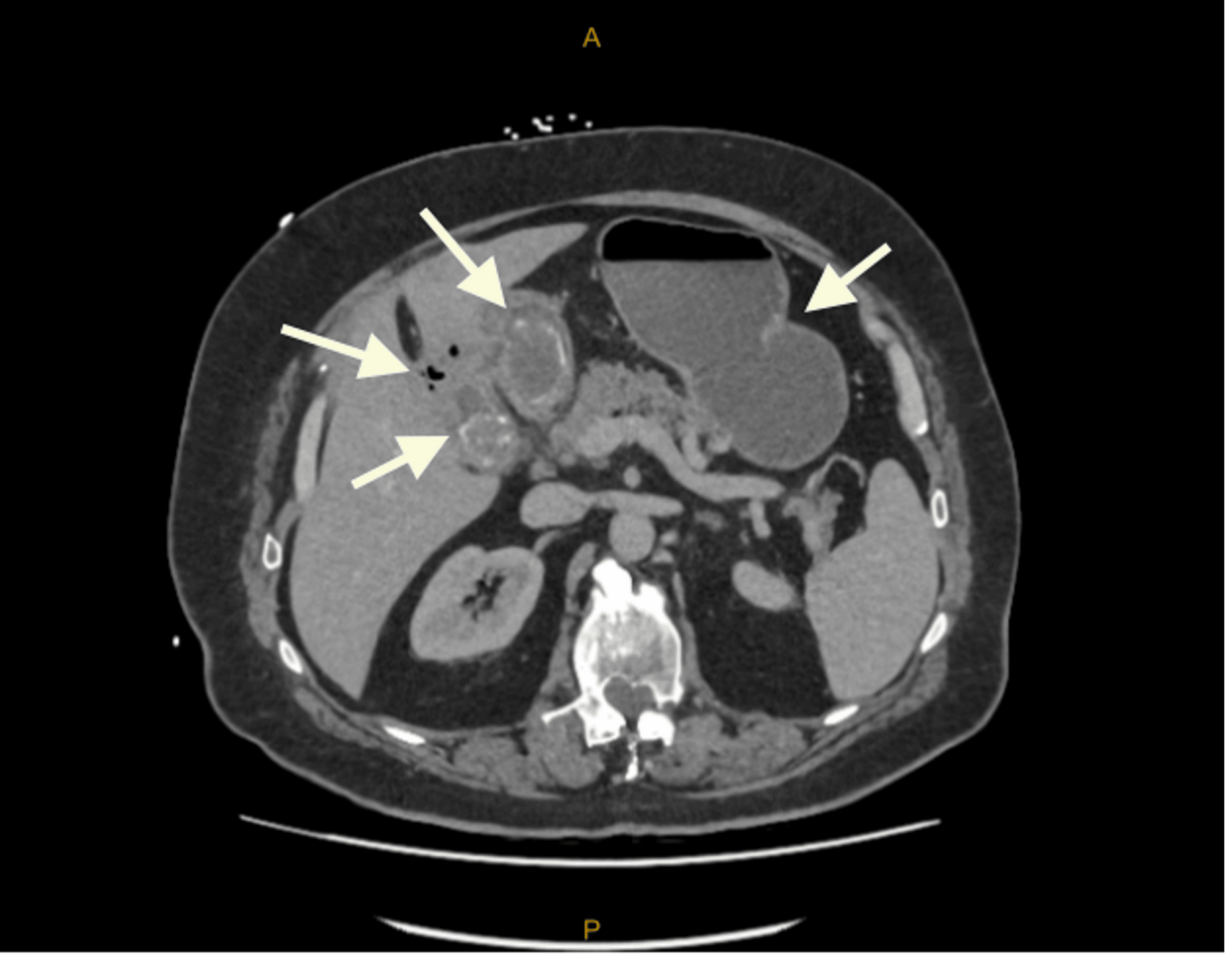
CTAP with IV contrast slices demonstrating gallbladder wall thickening and adjacent inflammatory change, fistulous tract between the pylorus/D1 segment and the gallbladder, 3.5 cm gallstone in proximal duodenum, associated pneumobilia and stomach distention (Case 3)

His CTAP showed marked gallbladder wall thickening and a fistulous tract between the pylorus/D1 segment of the duodenum and gallbladder (Figure 4).

There was a gallstone within the proximal duodenum measuring 3.5 cm and a further cholelithiasis within the gallbladder. There was also marked pneumobilia without biliary dilatation. The stomach appeared mildly distended. Her blood panel showed signs of partial biliary obstruction [1] (Table 3).

**Table 3:** Case 3 blood test results WCC: white cell count, ALT: alanine aminotransferase, AST: aspartate aminotransferase, ALP: alkaline phosphatase, GGT: gamma-glutamyl transferase

Blood test	Value	Normal range [2]
WCC	7.3	3.5-11.0 x 10^9/L
CRP	18	<3.0mg/L
Bilirubin	10	<20.0 mg/dL
ALT	39	<51 U/L
AST	19	<36 U/L
ALP	169	30-110 U/L
GGT	139	5-50 U/L

An NGT was inserted for gastric decompression, followed by transfer to the operating theatre.

Endoscopic retrieval of the stone was not attempted due to the stone's size (6 cm), and the patient underwent operative management as follows.

Initial laparoscopy revealed severely inflamed acute on chronic cholecystitis with adhesions. We performed adhesiolysis, laparoscopic cholecystectomy and intraoperative cholangiogram, which revealed filling defects in the bile duct. Choledochoscopy and transcystic common bile duct (CBD) clearance were performed with the removal of three CBD stones. This also visualised a large impacted gallstone in D1. A transverse excision of the bilioduodenal fistula was performed, and the stone was removed from the defect. This was primarily repaired with interrupted sutures. Additionally, we secured an omental patch over the defect. A 15Fr subhepatic BLAKE^TM^ (Ethicon, Sommerville, NJ, USA) drain was inserted to assess for biliary leak. All specimens were sent for histology, which later confirmed serosal inflammation and no features of malignancy.

The patient was managed in the ICU postoperatively and had an uneventful recovery. He did not require any haemodynamic support and was kept NBM for five days. He had a negative methylene blue test, which confirmed no leak. A follow-up gastroscopy at 4 months showed no strictures from the surgical site and an easily accessible D2.

Summary of Operative Steps

There was no attempt at endoscopic removal of the duodenal stone due to the large stone size and low chance of successful extraction. A cholecystectomy was performed. The cholecystoduodenal fistulae were resected, a large obstructing gallstone was removed, and the D1/D2 defect was repaired with primary closure and omental patch. A transcystic CBD exploration with choledochoscopy was performed, and obstructing bile duct stones were cleared.

## Discussion

Bouveret's syndrome is a rare complication of cholelithiasis, typically of stones with a diameter greater than 2.5 cm [1]. These stones can cause pressure necrosis of the gallbladder or bile duct wall and form fistulous tracts into the gastroenteric system, then they migrate and cause secondary gastric outlet or small bowel obstruction. Most stones are found in the terminal ileum (Barnard’s syndrome) and only 1-3% are found in the duodenum (Bouveret's syndrome) [3]. The larger the stone, the more proximal will be its site of impaction. As demonstrated in our series, the signs, symptoms and blood panels are non-specific to biliary tract disease, and diagnosis is usually achieved with CTAP. Rigler’s triad is present in 21% of patients, which demonstrates the presence of ectopic gallstones in the enteric system, upstream gastrointestinal tract dilatation and pneumobilia [4]. A defined fistulous tract between the biliary and enteric system may also be seen.

**Figure 5:**
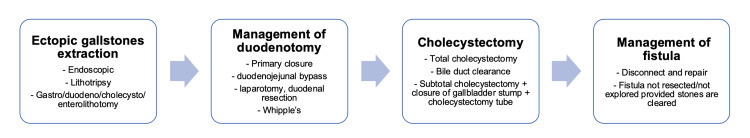
Summary of intraoperative management of Bouveret's syndrome

To date, no standardised guidelines exist, and management of gallstone ileus is largely individualised. The principles of management are ectopic gallstones extraction, cholecystectomy, fistula repair, and restoring enteric continuity (Figure 5).

Ectopic gallstones extraction

The most minimally invasive approach is via endoscopic stone extraction with nets or baskets and lithotripsy (mechanical, electrohydraulic (EHL), intracorporeal laser and extracorporeal shockwave therapy) [5]. Endoscopic lithotripsy prevents high-risk duodenotomy and is useful to break down stones for an easier distal enterotomy [6]. The success rate of stone fragmenting with EHL alone is 10%; however, when used in combination with balloon expansion or laser lithotripsy, it can achieve success rates between 45 to 100% [5]. The multimodal technique requires a high degree of endoscopist expertise. Importantly, the success of stone fragmentation is also dependent on the stone’s size, its composition and location for access.

As demonstrated in two of our cases, large stones are difficult to extract; however, the benefit of endoscopy in these complex cases is to allow direct visualisation of the fistula for surgical planning and relocation of the stone to a more favourable position for enterotomy. In Case 3, we did not attempt endoscopic management, knowing that the size of the stone would limit successful extraction. Furthermore, there was no available lithotripsy equipment in the facility.

Generally, if the endoscopic technique fails, gastro, duodeno or enterolithotomy can be performed. A longitudinal antimesenteric incision is commonly done, followed by transverse primary closure to avoid stenosis [7,8]. A much rarer technique, cholecystolithotomy, limits the need to disturb the fistula. This is done via a wide incision in the gallbladder fundus, stone extraction and gallbladder irrigation prior to primary closure with absorbable sutures [9,10].

Management of duodenotomy

Following stone extraction, we employ a step-up approach when closure is difficult or thought to be associated with the risk of leak. The following are our final recommendations.

The most commonly performed repair is via primary closure with a simple transverse repair. This technique is suitable for healthy tissue and smaller defects. A good approximation of tissue is paramount to prevent luminal narrowing.

In situations where the defect is in D1/D2, of large size and associated with risk of leak, we will consider a duodenal-jejunal bypass if the local environment allows it. This will include a distal gastrectomy and pancreas-preserving duodenectomy to remove the stricture and defect in D1/D2. The important principle for resection in this location is to avoid injury to the major and minor papillae. Surgeons need to be aware of the ampulla position in relation to the duodenotomy defect. A useful technique for ampulla protection is by introducing a Fogarty catheter into D2 via the cystic duct into the CBD and the major ampulla.

In a situation where the stone is impacted in D2/D3 and cannot be easily removed, we will recommend laparotomy and manual assessment to assess the mobility of the stone, relocating it to the stomach or towards the site of the defect. Surgeons must also assess tissue viability that might dictate debridement and local resection of the duodenum where the stone is impacted. From these assessments, a duodenal resection- either proximal, distal or total duodenectomy with preservation of the pancreas and restoration of continuity of duodenum or stomach +/- reimplantation of ampulla can be performed.

A final management approach is pancreaticoduodenectomy (Whipple’s). This repair technique is very rarely required, especially with a normal pancreas. It carries a high risk of complications, including pancreatic leak from soft pancreas and non-dilated pancreatic duct [11,12]. We would recommend against this procedure, given the benign nature of this disease and the available options listed above.

Cholecystectomy and management of cholecystoduodenal fistula

The next decision the surgeon must make is when or if to address the gallbladder and cholecystoduodenal fistula. The following are our suggestions.

We recommend the total cholecystectomy, disconnection and repair of the fistula.

If this is not possible, we recommend performing a subtotal cholecystectomy and removal of all gallbladder/ biliary stones. The gallbladder stump should be closed to avoid biliary leak. Our preference is to use a cholecystostomy tube to allow healing by secondary intention. The tissues in these situations are likely to be fragile and at risk of gallbladder and duodenal leak.

If the above procedures cannot be done laparoscopically, we would recommend converting to laparotomy and seeking help from hepatobiliary surgeons.

One or two-stage approach

The procedures discussed above can be performed at the index operation (“one-stage”) or offered as a delayed elective surgery (“two-stage”), or not at all. Traditionally, multiple literature reviews have had a consensus that primary repair of the fistula is unnecessary, as it may spontaneously close over time in up to 65% of cases [3,13]. Furthermore, a one-stage approach is associated with a high mortality rate (16.9%) compared to 11.7% for enterolithotomy alone. Some authors recommend limiting these additional procedures to patients who have retained gallstones, persistent symptoms and younger than 50 years old [14].

However, a more recent review by Rabie and Sokker found that in the last three decades, the mortality rate has been significantly reduced and is now similar (7.5% and 7.8% for the one-stage procedure and enterolithotomy alone, respectively) [9]. Nickel et al., in their review, proposed an algorithm that is based on the patient’s age and comorbidities [12]. For young, healthy patients with acceptable local tissue and absence of local inflammatory state, a one-stage procedure is advisable. This is to prevent secondary complications from recurrent attacks, which occur within the early postoperative phase, ranging from six weeks to six months from index presentation [15]. Eleven per cent of patients with persistent fistula may also experience cholangitis [16] and up to 10% require re-operation due to persistent biliary symptoms [17]. It is also important to note that a repeat laparotomy for a recurrent gallstone ileus carries the mortality risk up to 20% [17]. Therefore, for select patients, surgeons must weigh the risk of surgical complications against the risk of patients developing recurrent acute episodes.

In Case 1, following duodenotomy, we evaluated that the patient had remaining stones in the gallbladder. As the literature recommends, this requires exploration and management. Due to her morbid obesity, an open approach for cholecystectomy or gallstones removal would have been extremely challenging. Minimally invasive/ laparoscopic surgery enabled us to do a one-stage repair of the fistula, stone extraction from the duodenum, removal of remaining gallstones and subtotal cholecystectomy.

Similarly, in Case 3, the patient had stones in D1/D2, fistulae, and CBD, yet we were able to do full stone clearance via a difficult but one-staged surgical approach. The intraoperative findings for both cases dictate the necessity for complete stone retrieval, fistula repair and cholecystectomy for source control.

In Case 2, we did not perform a cholecystectomy. This is because the gallbladder was contracted and there were no remaining stones in the gallbladder. Additionally, there were dense adhesions to the hepatic flexure. All these factors in combination helped us decide that cholecystectomy was not necessary in the index operation. The patient can instead be monitored and be considered for a 2-stage operation if required.

Cholecystoduodenal fistula and malignancy

The incidence of cancer from this disease is worth noting. Large studies report a higher rate of cancer associated with biliary-enteric fistula (3-14%) [18] in comparison to the findings of carcinoma on gallbladder excised for lithiasis alone (0.3%) [19]. The associations are thought to be due to the anatomical disruption to the protective barrier of the papilla. The exposure of the biliary system to gut flora, constant back flow and chronic chemical irritation eventually results in epithelial cell changes and gallbladder malignancy [20]. For this reason, some authors recommend considering taking frozen sections of specimens that are suspicious for malignancy [19]. If a one-stage management approach involving cholecystectomy and fistula take-down was not possible, the patient should be referred to hepatobiliary services for follow-up surgical management or close surveillance. Due to the known cancer associations, we recommend ongoing clinical and imaging follow-up for patients with remnant gallbladder or fistula (as seen in Cases 1 and 2) to monitor for the development of gallbladder mass.

## Conclusions

Our case series depicts the variability of Bouveret’s syndrome presentation and management. A treatment algorithm is useful for rationalising options; however, surgeons have to be open-minded regarding a one-staged approach and offering cholecystectomy in the index operation for select patients. The considerations are disease-related, patient-related related and must be tailored to surgical expertise and equipment availability.
